# Fast identification of mineral inclusions in diamond at GSECARS using synchrotron X-ray microtomography, radiography and diffraction

**DOI:** 10.1107/S1600577519006854

**Published:** 2019-07-19

**Authors:** Michelle D. Wenz, Steven D. Jacobsen, Dongzhou Zhang, Margo Regier, Hannah J. Bausch, Przemyslaw K. Dera, Mark Rivers, Peter Eng, Steven B. Shirey, D. Graham Pearson

**Affiliations:** a Department of Earth and Planetary Sciences, Northwestern University, Technological Institute, 2145 Sheridan Road, Evanston, IL 60208, USA; b Hawaii Institute of Geophysics and Planetology, University of Hawaii, Honolulu, HI 96822, USA; c Department of Earth and Atmospheric Sciences, University of Alberta, Edmonton, Alberta T6G 2E9, USA; d Center for Advanced Radiation Sources, The University of Chicago, Chicago, IL 60637, USA; e Department of Terrestrial Magnetism, Carnegie Institution for Science, Washington DC 20015, USA

**Keywords:** diamond, minerals, microinclusions, computed microtomography, microdiffraction, radiography

## Abstract

Mineral inclusions in natural diamond are widely studied for the insight that they provide into the geochemistry and dynamics of the Earth’s interior. A major challenge in achieving thorough yet high rates of analysis of mineral inclusions in diamond derives from the micrometre-scale of most inclusions, often requiring synchrotron radiation sources for diffraction. Centering microinclusions for diffraction with a highly focused synchrotron beam cannot be achieved optically because of the very high index of refraction of diamond. A fast, high-throughput method for identification of micromineral inclusions in diamond has been developed at the GeoSoilEnviro Center for Advanced Radiation Sources (GSECARS), Advanced Photon Source, Argonne National Laboratory, USA. Diamonds and their inclusions are imaged using synchrotron 3D computed X-ray microtomography on beamline 13-BM-D of GSECARS. The location of every inclusion is then pinpointed onto the coordinate system of the six-circle goniometer of the single-crystal diffractometer on beamline 13-BM-C. Because the bending magnet branch 13-BM is divided and delivered into 13-BM-C and 13-BM-D stations simultaneously, numerous diamonds can be examined during coordinated runs. The fast, high-throughput capability of the methodology is demonstrated by collecting 3D diffraction data on 53 diamond inclusions from Juína, Brazil, within a total of about 72 h of beam time.

## Abstract 2

Microinclusions in diamond reveal geochemical signatures from the Earth’s deep mantle. A methodology is developed for non-destructive, high-throughput *in situ* characterization of mineral inclusions using synchrotron X-ray microtomography, radiography and diffraction at GSECARS, Sector 13 of the Advanced Photon Source.

## 1. Introduction   

Most diamonds are thought to crystalize in the mantle roots of the continental lithosphere (Stachel & Harris, 2008 ▸), whereas so-called super-deep diamonds and their inclusions are believed to crystalize in the convecting upper mantle, transition zone and even lower mantle (Nestola *et al.*, 2018 ▸; Palot *et al.*, 2016 ▸; Pearson *et al.*, 2014 ▸, 2003 ▸; Shirey *et al.*, 2013 ▸; Stachel *et al.*, 2005 ▸). Provided that the host diamonds are not cracked, minerals included within them are essentially encapsulated in an inert preservation vessel during eruption to the surface in kimberlitic magmas. The study of these micromineral inclusions provides insight into the geochemistry and dynamics of the Earth’s crust–mantle system from otherwise unattainable depths (Harte, 2011 ▸; Pearson *et al.*, 2014 ▸; Smith *et al.*, 2018 ▸). In the past, the study of diamond inclusions has been largely limited to destructive techniques, such as breaking the diamond to release inclusions or grinding away the host diamond to expose inclusions at the surface. In addition, the use of laboratory-source X-rays limits the minimum size of inclusion that can be identified by X-ray diffraction. Destructive extraction techniques have the inherent risk of losing or altering the inclusions, which are usually under remnant pressure inside the diamond host (Angel *et al.*, 2015 ▸). Thus, by studying these encapsulated inclusions using non-destructive methods, properties such as inclusion pressure, oxidation state, high-pressure phases and volatile content remain preserved.

To date, *in situ* identification of mineral inclusions in diamond via non-destructive methods remains challenging as the very high refractive index of diamond (*n* ≃ 2.4) hinders typical identification methods, such as optical microscopy or Raman spectroscopy, unless the inclusion is very close to a flat diamond surface. The high *n* of diamond also makes optical centering methods for single-crystal diffraction time consuming (Kunz *et al.*, 2002 ▸; Nestola *et al.*, 2012 ▸). In 2011, the first *in situ* crystal structure refinement of an inclusion in diamond was performed on an olivine crystal measuring ∼80 µm in the largest dimension using a sealed-tube Mo *K*α source (Nestola *et al.*, 2011 ▸). In that study, two large and parallel faces of the diamond facilitated optical centering of the inclusion. Subsequently, synchrotron radiation has been employed to carry out *in situ* structure refinements of clino­pyroxenes entrapped in diamond (Nestola *et al.*, 2016 ▸). Centering microinclusions inside highly irregular diamonds with an X-ray beam for diffraction can be accomplished by combining tomography with X-ray diffraction. Recently, this combined approach was employed by Nestola *et al.* (2012 ▸) using laboratory sources in Padova, Italy. The use of a laboratory source is ideal for the study of large (>50 µm) inclusions, but a method to quickly identify the multitude of smaller inclusions in large available suites of super-deep diamonds is required to obtain a more thorough sampling of inclusion mineralogy.

In this paper, we describe a fast, high-throughput and non-destructive methodology for identifying microinclusions in diamond as small as 10–20 µm in the maximum dimension by combining synchrotron microtomography with a newly developed radiography system now installed on the single-crystal diffraction beamline of the GeoSoilEnviro Center for Advanced Radiation Sources (GSECARS), Advanced Photon Source (APS), USA. Such a fast yet thorough method allows for all inclusions within the full volume of each diamond to be identified, thus allowing for a better relative modal proportion of inclusions to be obtained as smaller inclusions, which would be missed if only utilizing optical methods are not overlooked. In addition, the use of microtomography prior to diffraction provides detailed information on the integrity of the diamond host, revealing microcracks that may indicate the potential for metasomatic alteration of the inclusions. By identifying all microinclusions within a suite of super-deep diamonds, more information about the environment wherein superdeep diamonds form is obtained. The efficiency of the system is demonstrated by collecting 3D diffraction data from 53 inclusions in a total of 23 different diamonds from Juína, Brazil, all within a total of about 72 h of beam time. This result is unachievable by any other method.

## 2. Experimental   

### 2.1. Synchrotron microtomography   

**Figure 1:**
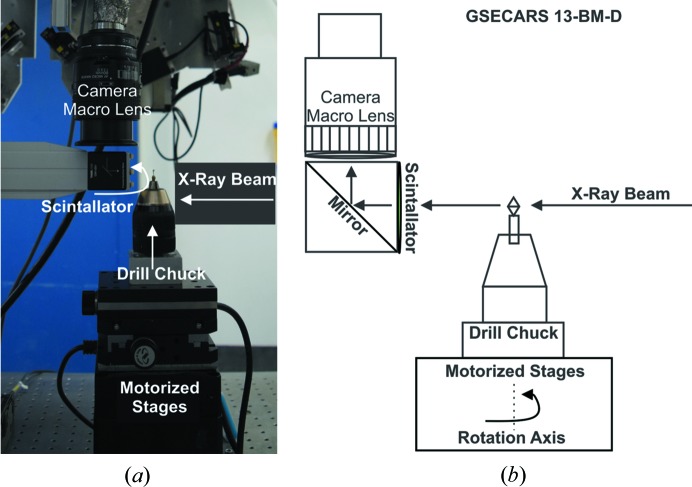
(*a*) Photograph and (*b*) schematic of the microtomography setup at 13-BM-D, GSECARS, Advanced Photon Source.

Synchrotron microtomography, used to physically locate mineral inclusions within the diamond, was conducted at GSECARS, beamline 13-BM-D, of the APS. The configuration of the 13-BM branch allows for simultaneous delivery of the X-ray beam to both 13-BM-D (microtomography) and 13-BM-C (single-crystal X-ray diffraction) beamlines. Thus, during coordinated runs the diamonds go directly from 13-BM-D to the newly developed 2D radiography and single-crystal diffraction system at 13-BM-C, which hosts the Partnership for eXtreme Xtallography (PX^2), a collaboration between the University of Hawaii and GSECARS, supported by the Consortium for Materials Properties Research in Earth Sciences (COMPRES). This facility is funded by COMPRES to advance crystallographic studies of minerals and materials under conditions of extreme pressures, temperatures and strain rates. A schematic diagram of the microtomography beamline illustrates the configuration used for super-deep diamonds, many of which have irregular shapes (Fig. 1 ▸). A monochromatic beam with an energy of 28.9 keV was chosen for this approach as this energy closely matches the operating energy of the 13-BM-C (28.6 keV) diffraction beamline. Due to the diffraction of the scintillator itself, Ce-doped LuAG, the exact operation energy of 13-BM-C could not be used because artifacts appeared in the tomographic reconstructions. Choosing a similar operating energy guarantees that all inclusions visible at the 13-BM-D beamline will also appear in the 2D radiography system at 13-BM-C. Due to the variability in both size and shape of super-deep diamonds, an adjustable field of view (FOV) is required to accurately map all inclusions. A typical FOV for large diamonds (∼6 mm) is around 8.70 mm by 5.44 mm. Collection times are on the order of 15 min per diamond, thus within a 24 h time period, full tomography on the entire volume of over 90 diamonds is achievable.

### 2.2. Synchrotron X-ray diffraction   

**Figure 2:**
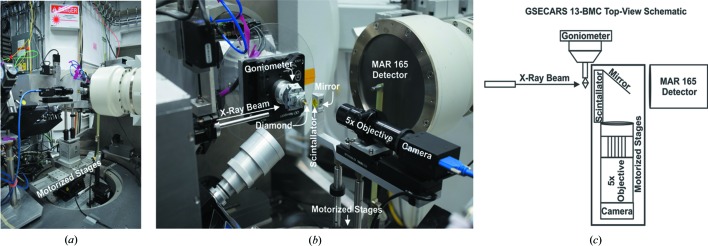
(*a*) Photograph of the entire portable radiograph attachment. (*b*) Close-up photograph of the portable radiography system. (*c*) Top-view schematic of the portable radiography system at 13-BM-C. The rectangle represents the motorized stage, yet also highlights the main components that make up the newly developed portable 2D radiography system available at 13-BM-C.

Single-crystal X-ray diffraction measurements were carried out at GSECARS, beamline 13-BM-C, of the APS. Combining microtomography and X-ray diffraction required the development of a portable 2D radiography attachment at 13-BM-C for the centering procedure. The components of this live radiography system consist of a scintillator, mirror, a 5× objective and a GigE camera. All components are mounted onto a motorized stage, which enables the radiography system to drive in and out of the X-ray beam (Fig. 2 ▸). Thus, the 2D radiography setup does not interfere with the six-circle goniometer during diffraction collection. EPICS *areaDetector* (Rivers, 2018 ▸; Rivers *et al.*, 2010 ▸) and *ImageJ* (Schneider *et al.*, 2012 ▸) are used to view the live radiograph while allowing for the constant normalization to a flat-field image.

The first step in setting up the diffraction experiment is to identify the rotation axis of the diffractometer and intersect the X-ray beam with it. The rotation axis of the diffractometer is set up in the horizontal direction, perpendicular to the incident X-ray (Zhang *et al.*, 2017 ▸). A focused X-ray beam with a full width at half-maximum of 12 µm (H) × 18 µm (V) is achieved by horizontal and vertical Kirkpatrick–Baez mirrors (Eng *et al.*, 1998 ▸). The rotation axis of the diffractometer is visualized by rotating a 25 µm-diameter tungsten wire. Once the tungsten wire ceases to precess during rotation, the tungsten wire coincides with the rotation axis. The X-ray beam vertical position is then adjusted until maximum absorption is detected. At this stage, the X-ray beam intersects the rotation axis of the diffractometer and the tungsten wire is then removed. The incident beam position is marked on the scintillator image with a virtual crosshair, which corresponds to the intersection of the rotation axis and the X-ray.

Obtaining a live radiograph image of the inclusion on the 13-BM-C diffraction beamline requires defocusing the X-ray beam to increase the FOV. An FOV of ∼100 µm (H) × 250 µm (V) is achieved by defocusing the Kirkpatrick–Baez mirrors, giving a magnified image of the inclusion for centering. Each inclusion is located by observing its absorption shadow in the radiograph image. Locating inclusions in this magnified FOV requires the use of high-resolution (4.5 mm per pixel) microtomography data obtained at beamline 13-BM-D prior to diffraction. Without microtomography data the process can take an hour or more to locate an inclusion within such a magnified FOV, whereas with the microtomography map it takes only a few minutes. Once an inclusion is found within the FOV and placed into the virtual crosshair, a rotation centering of the inclusion is performed in 5° steps, thus centering the inclusion on the rotation axis. Once the inclusion is properly centered, the X-ray beam then is refocused back to the virtual crosshair and the scintillator is driven out of the beam path. Single-crystal X-ray diffraction using a six-circle goniometer proceeds following standard X-ray diffraction protocols (Zhang *et al.*, 2017 ▸). It takes 5 min to collect a wide-scan diffraction image (rotation of 180°), and 30 min for step scan collections (steps in scan = 180, exposure time per degree = 1 s, rotation of 180°) using the MAR 165 CCD detector. The new Pilatus 1M detector with a 1 mm silicon sensor implemented in 2019 will speed these collections times up to a few minutes. Thus, 13-BM-C allows for fast diffraction analysis on a multitude of inclusions.

## 3. Results   

To demonstrate the capabilities of this new fast, high-throughput combined synchrotron microtomography and X-ray diffraction technique, a suite of 61 diamonds from Juína, Brazil, were studied. Microtomography data were collected on all 61 diamonds using a CMOS camera with 1920 × 1200 pixels, 4.5 µm pixel size on the sample, 1 s exposure time and 900 projections. All microtomography data on the 61 diamonds were collected within a 24 h period.

X-ray diffraction data, obtained using the newly developed live 2D radiograph centering technique at 13-BM-C, were collected on 53 inclusions found within 23 of these super-deep diamonds within a 72 h period. Sample-to-detector distances and tilt were calibrated using diffraction of LaB_6_. Single-crystal inclusion diffraction data were processed using the program *ATREX* (previously *GSE_ADA*; Dera *et al.*, 2013 ▸), which handles peak searching and fitting routines allowing for the generation of a peak list. To index the peaks, the peak list generated in *ATREX* was read into the program *Reciprocal Space Viewer* (Dera *et al.*, 2013 ▸), where peak indexing, orientation matrix determination and refinement of lattice parameters were performed. For powder inclusions as well as mixed-phase inclusions (inclusions with both powder and single-crystal phases), diffraction images were first integrated in *DIOPTAS* (Prescher & Prakapenka, 2015 ▸), a program designed specifically for handling large amounts of data collected at XRD beamlines in order to generate intensity versus 2θ plots. These 2θ plots were then imported into the program *GSAS-II* (Toby & Von Dreele, 2013 ▸) for further lattice parameter processing, indexing and refinement. Inclusions were ultimately identified via their lattice parameters.

Lattice parameters for the 53 inclusions are shown in Table 1 ▸. A summary of all minerals found from these diamonds is given in Table 2 ▸. The majority of inclusion phases form solid solutions. Thus, unit-cell volumes are dependent on both composition and remnant pressure. Phases along the hematite (Fe_2_O_3_) to ilmenite (FeTiO_3_) solid solution are referred to as titanohematite (Brown *et al.*, 1993 ▸). Phases along the magnetite (Fe_3_O_4_) to ulvöspinel (Fe_2_TiO_4_) solid solution are referred to as titanomagnetite (Bosi *et al.*, 2009 ▸). Olivine phases refer to those along the forsterite (Mg_2_SiO_4_) to fayalite (Fe_2_SiO_4_) solid solution series. Following standard mineralogical nomenclature, the (Mg,Fe)O oxides are classified such that samples containing <50 mol% FeO are referred to as ferropericlase and those with >50 mol% are magnesiowüstite (Jacobsen *et al.*, 2002 ▸; Prewitt & Downs, 1998 ▸). We note however that there is large uncertainty in the composition of such inclusions studied *in situ* using lattice parameters alone. For (Mg,Fe)O, assuming the variation of lattice parameter with *X*_Fe_ = ΣFe/(ΣFe + Mg) and an average bulk modulus *K*_T0_ of 160 GPa from the work of Jacobsen *et al.* (2002 ▸), the value of *X*_Fe_ would be underestimated by about 0.088 (or 8.8 mol% FeO) per GPa of remnant pressure.

It is interesting to note that the majority of the inclusions identified in our study are ferropericlase (Mg,Fe)O. Ferropericlase was reported previously as a predominate mineral in Juína diamonds and has often been associated with signifying a lower mantle origin (Anzolini *et al.*, 2019 ▸; Kaminsky *et al.*, 2009 ▸), whereas more non-pyrolitic Fe-rich (Mg,Fe)O inclusions are associated with conditions of diamond growth (Nimis *et al.*, 2019 ▸). Thomson *et al.* (2016 ▸) proposed that the presence of (Mg,Fe)O inclusions may be related to the reactions between the carbonatitic melt and reduced mantle peridotite. The range of intermediate and Fe-rich compositions reported in ferropericlase inclusions in diamond may represent different stages of the reaction (Thomson *et al.*, 2016 ▸). Because the numerous ferropericlase inclusions in the current suite of Juína diamonds are not associated with any high-pressure mineral inclusions, they are possibly associated with the melt reactions proposed by Thomson *et al.* (2016 ▸) and Nimis *et al.* (2019 ▸).

**Figure 3:**
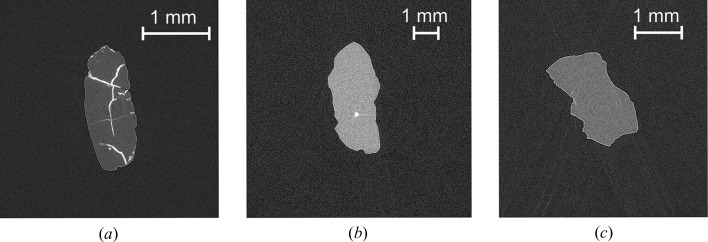
(*a*) Tomographic slice of diamond 6b_24 exhibiting multiple cracks. (*b*) Tomographic slice of diamond 6b_09 with a high-absorbing goethite inclusion, FeOOH. (*c*) Tomographic slice of diamond 6b_56 with a less absorbing silicate inclusion olivine, (Mg,Fe)_2_SiO_4_.

The advantages of high-resolution microtomography extend beyond the X-ray centering procedure as this technique also reveals information on both the number and quality of the inclusions. Super-deep diamonds exhibit rough irregular shapes as well as different surface textures, which often preclude optical observation. Tomography reveals all inclusions, even those not visible under optical microscopes, and also provides a way of checking that the inclusion is pristine. Super-deep diamonds experience extreme stresses and therefore some exhibit microcracks only visible via tomography [Fig. 3 ▸(*a*)]. These cracks often lead up to or surround an inclusion, which indicates that an inclusion may have interacted with kimberlitic magma or has cracked as a result of a difference in the elastic relaxation between the inclusion and the host diamond. Such information is lost when inclusions are extracted and yet this information is important when considering how representative an inclusion is of the mantle.

The capability to pre-screen inclusions also exists with microtomography. The reconstructed slices, *i.e.* maps of the absorption, provide the ability to distinguish between high and low X-ray absorbing inclusions. Differences in absorption indicate compositional differences owing to the mean atomic weight differences. Inclusions with high iron contents such as ferropericlase, (Mg,Fe)O, appear brighter in the tomographic slice than inclusions with lower absorbing material such as silicates or graphite [Figs. 3 ▸(*b*) and 3(*c*)].

## 4. Conclusions   

**Figure 4:**
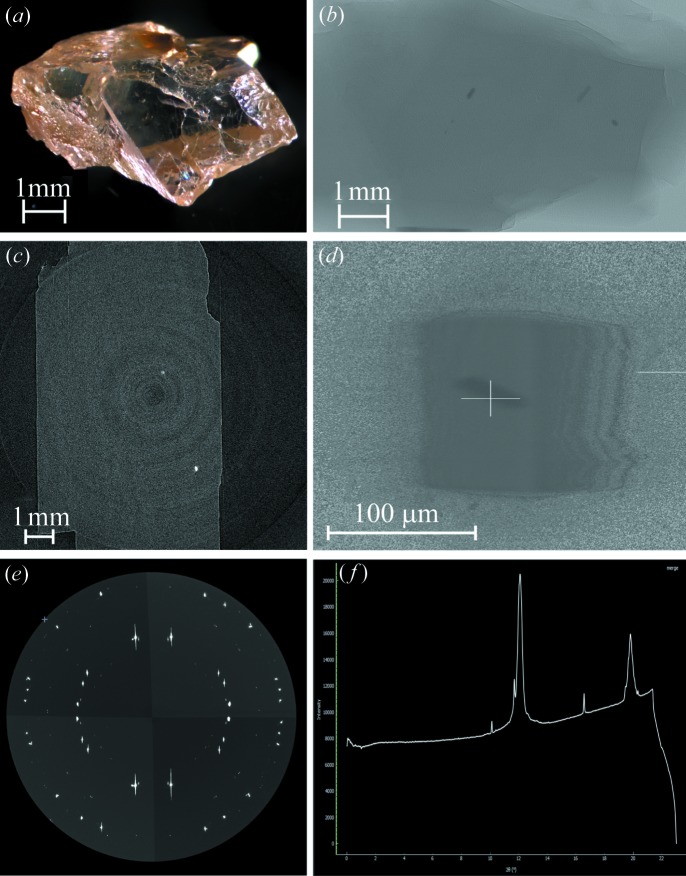
(*a*) Photomicrograph of Juína diamond 6B_06. (*b*) Radiograph of diamond 6B_06 taken at 13-BM-D. (*c*) Reconstructed slice of diamond 6B_06 from 13-BM-D data. Rings observed in the image are artifacts. (*d*) Radiograph of one of the ferropericlase inclusions in (*b*) taken at 13-BM-C. (*e*) Wide-scan (180° rotation) XRD image of a ferropericlase inclusion in diamond 6B_06 shown in (*d*). (*f*) Integrated diffraction pattern of ferropericlase inclusion in diamond 6b_06, image produced using *DIOPTAS* (Prescher & Prakapenka, 2015 ▸).

A fast, high-throughput method developed at GSECARS (Sector 13) of the APS provides the opportunity for dozens of inclusions within a diamond suite to be identified within days (Fig. 4 ▸). The high quality of both the microtomography and X-ray data reveals clues to the history of the inclusion. Identifying all inclusions within a diamond suite garners insight into the composition and geochemical cycling of Earth’s dynamic mantle that remains unattainable with small sample sets. Serving as the only samples from such depths, diamond inclusions hold the key to unlocking the secrets of Earth’s mantle.

## Figures and Tables

**Table 1:** Symmetry-constrained lattice parameters of 53 inclusions identified in a suite of diamonds from the São Luiz locality in Juína, Brazil. Single-crystal inclusions denoted by *, the rest of the inclusions are powder

Inclusion	*a* (Å)	*b* (Å)	*c* (Å)	α (°)	β (°)	γ (°)	Volume (Å^3^)	Symmetry constraints	Mineral
6b_04b*	8.509 (2)	8.509 (2)	8.509 (2)	90	90	90	616.0 (2)	Cubic	Titanomagnetite Fe_1+*x*_(Fe_2–2*x*_Ti_*x*_)O_4_
6b_04b2*	4.255 (1)	4.255 (1)	4.255 (1)	90	90	90	77.1 (6)	Cubic	Ferropericlase (Mg_*x*_,Fe_1–*x*_)O
6b_05a*	4.246 (4)	4.246 (4)	4.246 (4)	90	90	90	76.6 (2)	Cubic	Ferropericlase (Mg_*x*_,Fe_1–*x*_)O
6b_05b*	4.255 (2)	4.255 (2)	4.255 (2)	90	90	90	77.0 (2)	Cubic	Ferropericlase (Mg_*x*_,Fe_1–*x*_)O
6b_05c*	4.259 (1)	4.259 (1)	4.259 (1)	90	90	90	77.3 (1)	Cubic	Magnesiowüstite (Mg_1–*x*_,Fe_*x*_)O
6b_05d*	4.262 (2)	4.262 (2)	4.262 (2)	90	90	90	77.4 (2)	Cubic	Magnesiowüstite (Mg_1–*x*_,Fe_*x*_)O
6b_05e*	4.251 (2)	4.251 (2)	4.251 (2)	90	90	90	77.4 (2)	Cubic	Ferropericlase (Mg_*x*_,Fe_1–*x*_)O
6b_06a*	4.276 (2)	4.276 (2)	4.276 (2)	90	90	90	78.2 (1)	Cubic	Magnesiowüstite (Mg_1–*x*_,Fe_*x*_)O
6b_06b*	4.271 (7)	4.271 (7)	4.271 (7)	90	90	90	77.9 (1)	Cubic	Magnesiowüstite (Mg_1–*x*_,Fe_*x*_)O
6b_07a	2.868 (9)	2.868 (9)	2.868 (9)	90	90	90	23.6 (2)	Cubic	Fe (b.c.c.) with some alloy
6b_07b	2.868 (5)	2.868 (5)	2.868 (5)	90	90	90	23.6 (1)	Cubic	Fe (b.c.c.) with some alloy
6b_07c*	4.276 (2)	4.276 (2)	4.276 (2)	90	90	90	78.2 (1)	Cubic	Magnesiowüstite (Mg_1–*x*_,Fe_*x*_)O
6b_07c2*	8.442 (5)	8.442 (5)	8.442 (5)	90	90	90	601.7 (3)	Cubic	Titanomagnetite Fe_1+*x*_(Fe_2–2*x*_Ti_*x*_)O_4_
6b_07d*	4.204 (5)	4.204 (5)	4.204 (5)	90	90	90	75.3 (3)	Cubic	Ferropericlase (Mg_*x*_,Fe_1–*x*_)O
6b_07d2*	8.511 (1)	8.511 (1)	8.511 (1)	90	90	90	601.7 (3)	Cubic	Titanomagnetite Fe_1+*x*_(Fe_2–2*x*_Ti_*x*_)O_4_
6b_07e*	4.320 (7)	4.320 (7)	4.320 (7)	90	90	90	81.0 (4)	Cubic	Wüstite FeO
6b_07e2*	8.490 (5)	8.490 (5)	8.490 (5)	90	90	90	612.0 (2)	Cubic	Titanomagnetite Fe_1+*x*_(Fe_2–2*x*_Ti_*x*_)O_4_
6b_08c*	5.083 (1)	5.083 (1)	5.083 (1)	90	90	120	314.6 (3)	Hexagonal	Ilmenite FeTiO_3_
6b_09	4.640 (6)	10.005 (9)	3.028 (3)	90	90	90	140.6 (2)	Orthorhombic	Goethite (FeOOH)
6b_10b	5.032 (1)	5.032 (1)	13.759 (3)	90	90	120	301.7 (1)	Hexagonal	Hematite Fe_2_O_3_
6b_10c	5.140 (3)	5.140 (3)	13.420 (2)	90	90	120	307.5 (2)	Hexagonal	Titanohematite [*x*FeTiO_3_(1–*x*)Fe_2_O_3_]
6b_11b*	8.396 (2)	8.396 (2)	8.396 (2)	90	90	90	591.8 (2)	Cubic	Magnetite Fe_3_O_4_
6b_12a*	4.273 (2)	4.273 (2)	4.273 (2)	90	90	90	78.0 (3)	Cubic	Magnesiowüstite (Mg_1–*x*_,Fe_*x*_)O
6b_12b*	4.270 (1)	4.270 (1)	4.270 (1)	90	90	90	77.9 (2)	Cubic	Magnesiowüstite (Mg_1–*x*_,Fe_*x*_)O
6b_12c*	4.280 (9)	4.280 (9)	4.280 (9)	90	90	90	78.4 (5)	Cubic	Magnesiowüstite (Mg_1–*x*_,Fe_*x*_)O
6b_12d*	4.274 (3)	4.274 (3)	4.274 (3)	90	90	90	78.1 (5)	Cubic	Magnesiowüstite (Mg_1–*x*_,Fe_*x*_)O
6b_17b*	4.270 (1)	4.270 (1)	4.270 (1)	90	90	90	77.8 (2)	Cubic	Magnesiowüstite (Mg_1–*x*_,Fe_*x*_)O
6b_17c*	4.285 (1)	4.285 (1)	4.285 (1)	90	90	90	78.7 (5)	Cubic	Magnesiowüstite (Mg_1–*x*_,Fe_*x*_)O
6b_21c*	4.279 (2)	4.279 (2)	4.279 (2)	90	90	90	78.3 (2)	Cubic	Magnesiowüstite (Mg_1–*x*_,Fe_*x*_)O
6b_21c2	8.405 (2)	8.405 (2)	8.405 (2)	90	90	90	593.8 (2)	Cubic	Titanomagnetite Fe_1+*x*_(Fe_2–2*x*_Ti_*x*_)O_4_
6b_23*	4.232 (8)	4.232 (8)	4.232 (8)	90	90	90	75.8 (3)	Cubic	Ferropericlase (Mg_*x*_,Fe_1–*x*_)O
6b_29a*	4.261 (2)	4.261 (2)	4.261 (2)	90	90	90	77.8 (2)	Cubic	Magnesiowüstite (Mg_1–*x*_,Fe_*x*_)O
6b_29b*	4.253 (1)	4.253 (1)	4.253 (1)	90	90	90	76.9 (1)	Cubic	Ferropericlase (Mg_*x*_,Fe_1–*x*_)O
6b_34a*	4.243 (2)	4.243 (2)	4.243 (2)	90	90	90	76.4 (2)	Cubic	Ferropericlase (Mg_*x*_,Fe_1–*x*_)O
6b_34b*	4.245 (1)	4.245 (1)	4.245 (1)	90	90	90	76.5 (1)	Cubic	Ferropericlase (Mg_*x*_,Fe_1–*x*_)O
6b_34c*	4.252 (2)	4.252 (2)	4.252 (2)	90	90	90	76.8 (2)	Cubic	Ferropericlase (Mg_*x*_,Fe_1–*x*_)O
6b_37a	4.254 (2)	4.254 (2)	4.254 (2)	90	90	90	77.0 (2)	Cubic	Ferropericlase (Mg_*x*_,Fe_1–*x*_)O
6b_37a2	8.379 (2)	8.379 (2)	8.379 (2)	90	90	90	588.0 (4)	Cubic	Magnetite Fe_3_O_4_
6b_39a	5.037 (5)	5.037 (5)	13.769 (1)	90	90	120	302.5 (4)	Hexagonal	Titanohematite [*x*FeTiO_3_(1–*x*)Fe_2_O_3_]
6b_39b	5.038 (7)	5.038 (7)	13.761 (1)	90	90	120	302.5 (5)	Hexagonal	Titanohematite [*x*FeTiO_3_(1–*x*)Fe_2_O_3_]
6b_46*	11.584 (3)	11.584 (3)	11.584 (3)	90	90	90	1554.4 (6)	Cubic	Almandine Fe_3_Al_2_(SiO_4_)_3_
6b_48b	4.744 (4)	10.185 (1)	5.978 (7)	90	90	90	288.8 (6)	Orthorhombic	Olivine (Mg_*x*_,Fe_2–*x*_)SiO_4_
6b_53*	4.246 (1)	4.246 (1)	4.246 (1)	90	90	90	76.5 (1)	Cubic	Ferropericlase (Mg_*x*_,Fe_1–*x*_)O
6b_54b*	6.609 (2)	6.609 (2)	6.001 (3)	90	90	90	262.1 (2)	Tetragonal	Zircon ZrSiO_4_
6b_56a	4.758 (7)	10.209 (6)	5.972 (7)	90	90	90	290.1 (4)	Orthorhombic	Olivine (Mg_*x*_,Fe_2–*x*_)SiO_4_
6b_56b	4.759 (8)	10.209 (8)	5.976 (1)	90	90	90	290.4 (5)	Orthorhombic	Olivine (Mg_*x*_,Fe_2–*x*_)SiO_4_
6b_56b2	8.394 (6)	8.394 (6)	8.394 (6)	90	90	90	591.4 (1)	Cubic	Magnetite Fe_3_O_4_
6b_56c	4.754 (1)	10.205 (7)	5.978 (1)	90	90	90	290.0 (6)	Orthorhombic	Olivine (Mg_*x*_,Fe_2–*x*_)SiO_4_
6b_56d	4.756 (1)	10.206 (1)	5.981 (1)	90	90	90	290.3 (6)	Orthorhombic	Olivine (Mg_*x*_,Fe_2–*x*_)SiO_4_
5a_09a	5.077 (3)	5.077 (3)	13.894 (4)	90	90	120	310.1 (2)	Hexagonal	Titanohematite [*x*FeTiO_3_(1–*x*)Fe_2_O_3_]
5a_09b	5.069 (2)	5.069 (2)	13.931 (5)	90	90	120	310.1 (2)	Hexagonal	Titanohematite [*x*FeTiO_3_(1–*x*)Fe_2_O_3_]
5a_10f*	4.281 (8)	4.281 (8)	4.281 (8)	90	90	90	78.5 (4)	Cubic	Magnesiowüstite (Mg_1–*x*_,Fe_*x*_)O
5a_20c*	4.245 (9)	4.245 (9)	4.245 (9)	90	90	90	76.47 (7)	Cubic	Ferropericlase (Mg_*x*_,Fe_1–*x*_)O

**Table 2:** Summary of all minerals found in the 23 diamonds from the São Luiz locality in Juína, Brazil

Mineral	No. of inclusions
Ferropericlase (Mg_*x*_,Fe_1–*x*_)O	13
Magnesiowüstite (Mg_1–*x*_,Fe_*x*_)O	14
Wüstite FeO	1
Magnetite (Fe_3_O_4_)	3
Titanomagnetite Fe_1+*x*_(Fe_2–2*x*_Ti_*x*_)O_4_	5
Hematite (Fe_2_O_3_)	1
Titanohematite [*x*FeTiO_3_(1–*x*)Fe_2_O_3_]	5
Olivine (Mg_*x*_,Fe_2–*x*_)SiO_4_	5
Iron (Fe)	2
Goethite (FeOOH)	1
Ilmenite (FeTiO_3_)	1
Garnet Fe_3_Al_2_(SiO_4_)_3_	1
Zircon (ZrSiO_4_)	1
